# Case report: Preclinical efficacy of NEDD8 and proteasome inhibitors in patient-derived models of signet ring high-grade mucinous colorectal cancer from a Lynch syndrome patient

**DOI:** 10.3389/fonc.2023.1130852

**Published:** 2023-02-02

**Authors:** Erica Torchiaro, Consalvo Petti, Sabrina Arena, Francesco Sassi, Giorgia Migliardi, Alfredo Mellano, Roberta Porporato, Marco Basiricò, Loretta Gammaitoni, Enrico Berrino, Monica Montone, Giorgio Corti, Giovanni Crisafulli, Caterina Marchiò, Alberto Bardelli, Enzo Medico

**Affiliations:** ^1^ Candiolo Cancer Institute, Fondazione del Piemonte per l'Oncologia (FPO) - IRCCS, Candiolo, Italy; ^2^ Department of Oncology, University of Torino, Candiolo, Italy; ^3^ Department of Medical Sciences, University of Turin, Torino, Italy; ^4^ IFOM ETS, The AIRC Institute of Molecular Oncology, Milano, Italy

**Keywords:** Lynch syndrome, mucinous colorectal cancer, signet ring cells, NEDD8 pathway inhibition, proteasome inhibition, preclinical study

## Abstract

High-grade mucinous colorectal cancer (HGM CRC) is particularly aggressive, prone to metastasis and treatment resistance, frequently accompanied by “signet ring” cancer cells. A sizeable fraction of HGM CRCs (20-40%) arises in the context of the Lynch Syndrome, an autosomal hereditary syndrome that predisposes to microsatellite instable (MSI) CRC. Development of patient-derived preclinical models for this challenging subtype of colorectal cancer represents an unmet need in oncology. We describe here successful propagation of preclinical models from a case of early-onset, MSI-positive metastatic colorectal cancer in a male Lynch syndrome patient, refractory to standard care (FOLFOX6, FOLFIRI-Panitumumab) and, surprisingly, also to immunotherapy. Surgical material from a debulking operation was implanted in NOD/SCID mice, successfully yielding one patient-derived xenograft (PDX). PDX explants were subsequently used to generate 2D and 3D cell cultures. Histologically, all models resembled the tumor of origin, displaying a high-grade mucinous phenotype with signet ring cells. For preclinical exploration of alternative treatments, in light of recent findings, we considered inhibition of the proteasome by bortezomib and of the related NEDD8 pathway by pevonedistat. Indeed, sensitivity to bortezomib was observed in mucinous adenocarcinoma of the lung, and we previously found that HGM CRC is preferentially sensitive to pevonedistat in models with low or absent expression of cadherin 17 (CDH17), a differentiation marker. We therefore performed IHC on the tumor and models, and observed no CDH17 expression, suggesting sensitivity to pevonedistat. Both bortezomib and pevonedistat showed strong activity on 2D cells at 72 hours and on 3D organoids at 7 days, thus providing valid options for *in vivo* testing. Accordingly, three PDX cohorts were treated for four weeks, respectively with vehicle, bortezomib and pevonedistat. Both drugs significantly reduced tumor growth, as compared to the vehicle group. Interestingly, while bortezomib was more effective *in vitro*, pevonedistat was more effective *in vivo*. Drug efficacy was further substantiated by a reduction of cellularity and of Ki67-positive cells in the treated tumors. These results highlight proteasome and NEDD8 inhibition as potentially effective therapeutic approaches against Lynch syndrome-associated HGM CRC, also when the disease is refractory to all available treatment options.

## Introduction

Colorectal cancer (CRC) is one of the most common causes of cancer-related death in the world (1). High-grade mucinous (HGM) CRC occurs in about 10-20% of cases (2) and is characterized by abundant extracellular mucin that accounts for at least 50% of the tumor volume. Although actively secreting mucins, HGM CRC cells are poorly differentiated, and indicate worse prognosis (3). This counterintuitive property is further exacerbated by the presence of “signet ring” cells, that do not interact with each other and contain a large vacuole filled with mucus (4). Signet ring CRC (SR-CRC) is more frequent in young patients (5) (6), and is endowed with marked metastatic propensity (7). HGM/SR-CRC is more frequently found in the proximal colon (8) and typically diagnosed in advanced stage.

HGM/SR-CRC frequently displays microsatellite instability (MSI) and the consequent propensity to accumulate mutations, leading to genetic evolution. Interestingly, 20-40% of mucinous CRCs arise at young age in the context of the Lynch Syndrome (9), an autosomal hereditary syndrome that predisposes to MSI CRC (2). Additional molecular features of HGM/SR-CRC include mutations in key genes of the RAS/MAPK (10) and PI3K/AKT/mTOR pathways (11), and overexpression of specific mucin genes, like *MUC2* and *MUC5AC* (11).

Until now no specific clinical guidelines have been developed for mucinous CRC patients, therefore they undergo standard CRC treatments including FOLFOX (leucovorin, fluorouracil, oxaliplatin), XELOX (capecitabine and oxaliplatin), and FOLFIRI (folic acid, fluorouracil and irinotecan) (12). Checkpoint blockade immunotherapy is an additional option for patients with MSI-positive disease (13). Typically, SR-CRC patients are less responsive to treatment, with shorter overall survival (3, 14). Consequently, new therapeutic options represent a still unmet clinical need.

The most effective way to explore alternative antineoplastic therapies relies on derivation and testing of patient-derived models, such as cell lines, organoids (PDOs) and xenografts (PDXs) (15, 16). However, an extensive internet and literature search for SR-CRC patient-derived models was unsuccessful, reflecting the need to obtain such models for preclinical explorations. We therefore propagated and extensively characterized 2D and 3D cell cultures and patient-derived xenografts (PDXs) from a case of early onset, MSI-positive metastatic SR-CRC in a Lynch syndrome patient, unresponsive to standard care (FOLFOX6, FOLFIRI-Panitumumab) and, surprisingly, also to immunotherapy with nivolumab.

We have previously found that inhibition of the NEDD8 pathway by the small molecule pevonedistat, also known as MLN4924 (17) is effective on mucinous CRC *in vitro* and *in vivo*, in cell lines and PDXs (18). NEDD8 is an ubiquitin-like peptide that, when conjugated to target proteins, modulates their activity. Major targets of neddylation are the ubiquitin ligases of the cullin-ring family, that in turn ubiquitinate and direct to the proteasome specific subsets of target proteins (19). Interestingly, the proteasome inhibitor bortezomib displayed efficacy in invasive lung adenocarcinoma patients only in the case of mucinous tumors (20). We therefore considered pevonedistat and bortezomib as promising candidate drugs to be tested in the newly derived SR-CRC cells and PDXs.

## Case description

**Figure 1:**
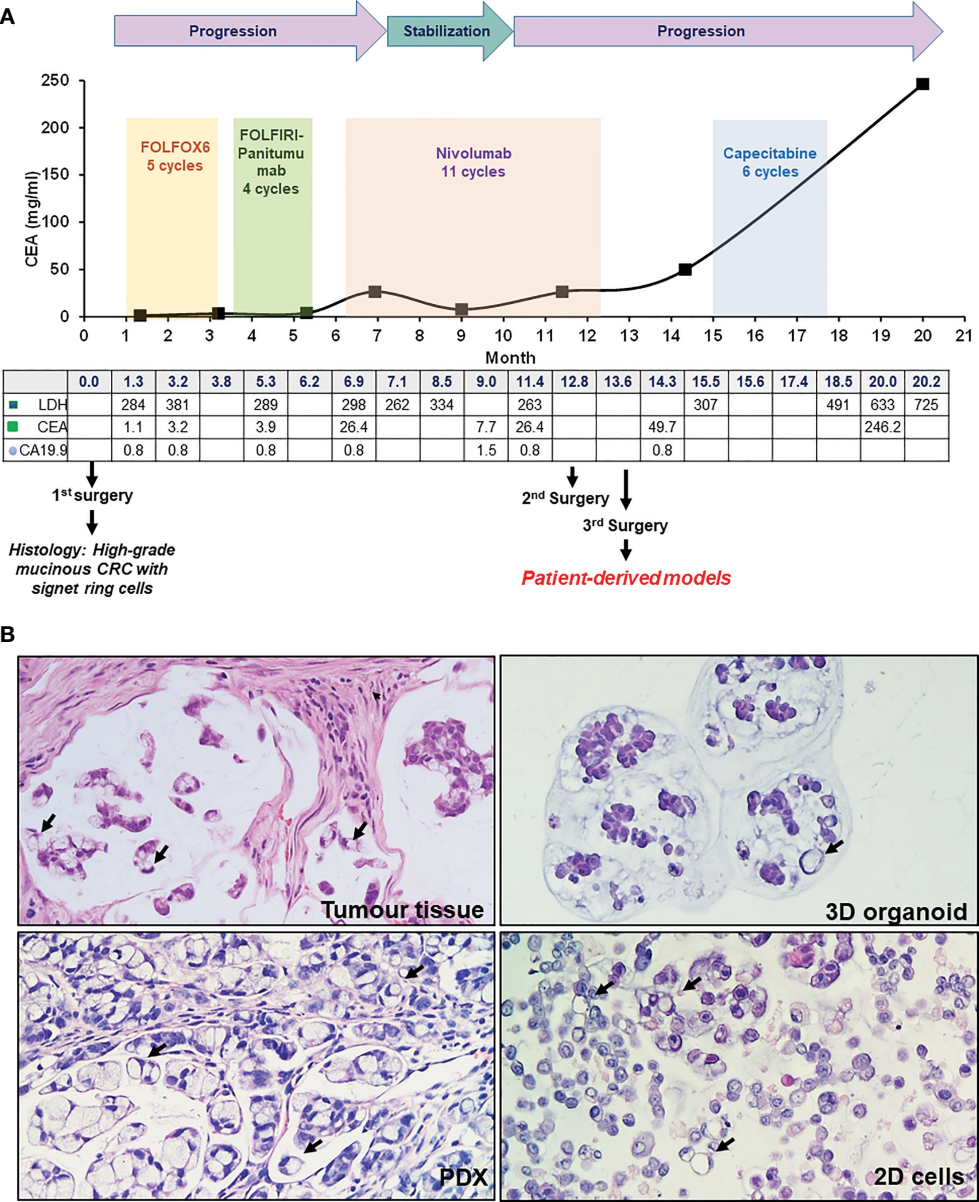
Patient case history and tumor histology. **(A)** Case history, outlining clinical progression, therapy and surgery history, and blood marker profiles. **(B)** H&E staining of patient tumor tissue (third surgery), PDX, organoid and 2D cells culture, as indicated. Signet ring cells are highlighted by black arrows (20X magnification).

The clinical history of this case is summarized in **Figure 1A**. A 26-year-old male patient presented with abdominal pain and underwent cholecystectomy. After one month, a CT scan highlighted a neoplastic lesion in the right colon with multiple mesenteric lymphadenopathies and nodules of peritoneal carcinosis. Histological analysis of a colonoscopy biopsy revealed SR-CRC. The patient immediately underwent right hemicolectomy, lymphadenectomy and exeresis of carcinosis nodes. Histological evaluation of the surgical specimens confirmed the diagnosis of SR-CRC in all samples. The molecular pathology report described a positive MSI status with negativity of tumor tissue for PMS2 and MLH1, no mutations in *KRAS*, *NRAS* and *BRAF* and p.His1047Arg mutation in exon 20 of the *PIK3CA* gene. Germline analysis revealed heterozygous frameshift mutation of *MLH1*, consistent with a Lynch syndrome diagnosis. Four weeks after surgery, new solid tissue formations and suspect lymph nodes were detected by CT scan, while blood levels of CEA were still low (1.1 mg/ml). After 5 cycles of FOLOFOX6, a CT scan showed minimal dimensional increase in different lesions, and CEA increased to 3.2 mg/ml. Considering the *KRAS* wild type status, FOLFIRI plus panitumumab was chosen as second line treatment. After 4 cycles, a CT showed new peritoneal lesions and enlargement of old formations, with CEA = 3.9 mg/ml, which led to further treatment change to nivolumab. After an initial disease stabilization, by the 11^th^ nivolumab cycle a drastic increment of CEA (26.4 mg/ml) was observed, with PET and CT detecting substantial increment of lesions size. An explorative laparotomy was performed together with Pressurized Intraperitoneal Aerosol Chemotherapy (PIPAC) with oxaliplatin. After this, a third cytoreductive surgery was performed. Patient-derived models were obtained from this surgery. Subsequently, the patient underwent adjuvant chemotherapy with capecitabine. Due to further disease progression, a palliative cytoreductive surgery was performed and eventually the patient passed away. In summary, this case of SR-CRC did not display clinical response to any line of treatment.

## Results

### Derivation and characterization of PDX and *in vitro* models

**Figure 2:**
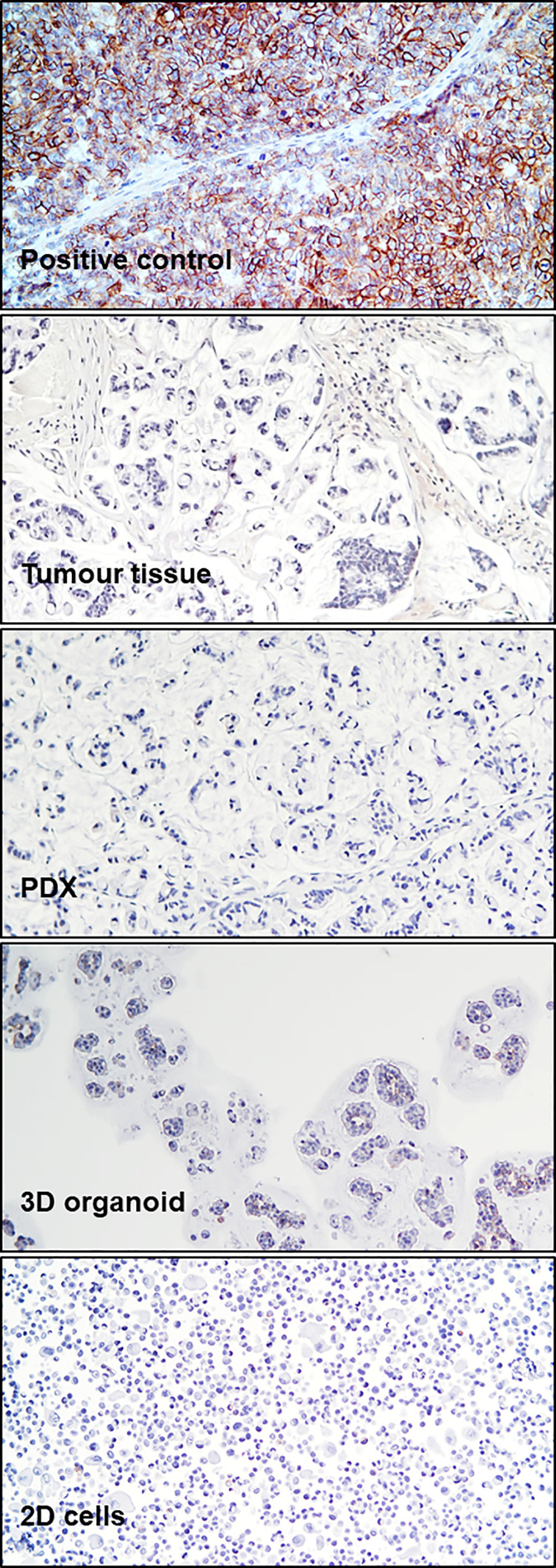
CDH17 Immunohistochemistry. Immunohistochemical staining for CDH17 in a positive control (xenograft SNU1746 cell line) and in patient tumor tissue (third surgery), PDX, 3D organoids, and 2D cells, as indicated.

To analyze tumor tissue morphology, hematoxylin/eosin staining (H&E) was performed, highlighting a highly mucinous tumor with abundant signet ring cells and conspicuous stromal infiltration (**Figure 1B**). A PDX line was generated from a colon lesion obtained from the third surgery. Subsequently, a 3D organoid line and a 2D adherent cell line were derived from the PDX (see Methods). Both 2D and 3D cultures survived more than one freeze/thaw cycles, and displayed massive mucus production in the culture medium. H&E staining of cell and organoid cytoclots showed abundant signet ring cells and mucus, similar to the tumor of origin (**Figure 1B**). All patient derived models, together with the tumor of origin, stained negative for cadherin 17 (CDH17, from Abnova), indicating a poorly differentiated tumor and potential sensitivity to pevonedistat (18) (**Figure 2**). To verify if the models maintained the molecular profile of the tumor of origin, and to search for actionable molecular alterations, deep sequencing of a 161-gene panel (ThermoFisher Oncomine Comprehensive Assay v.3) was performed on germline (DNA from white blood cells), tumor (from third surgery) and models, setting the variant allele frequency (VAF) for somatic mutation detection at 0.02. Germline analysis confirmed the MLH1 p Glu23Glyfs*8 frameshift mutation due to the insertion of an additional G in a stretch of five Gs. We observed strong concordance between the tumor tissue and all derived models, unfortunately with no targetable alteration. All samples (tumor tissue, 2D cells, 3D organoids and PDX) carried KRAS p.Gly13Asp mutation, most probably selected during the panitumumab treatment (21), CTNNB1 p.Ser45Phe and GNAS p.Arg201His. The PDX also showed a subclonal ERBB2 variant (p.Arg896His) and a frameshift deletion of *ARID1A* gene (p.Ile1816fs). Interestingly, no PIK3CA mutations were detected, highlighting heterogeneity or molecular evolution of the disease after the first surgery. The observed beta-catenin gain-of-function mutation, CTNNB1 Ser45Phe, is quite common in CRC patients (22), and could possibly be exploited in the future for combined treatments (data here described are reported in **Table 1 in Supplementary**). To further search for possible targets, whole exome sequencing was performed on tumor and germline DNA. All previously found mutations, including the germline MLH1 variant, were confirmed, but again no targetable mutations were found. A copy number gain was found in the long arm of chromosome 1, frequently observed in CRC (23), but with no current clinical implications.

### Preclinical evaluation of drug response

**Figure 3:**
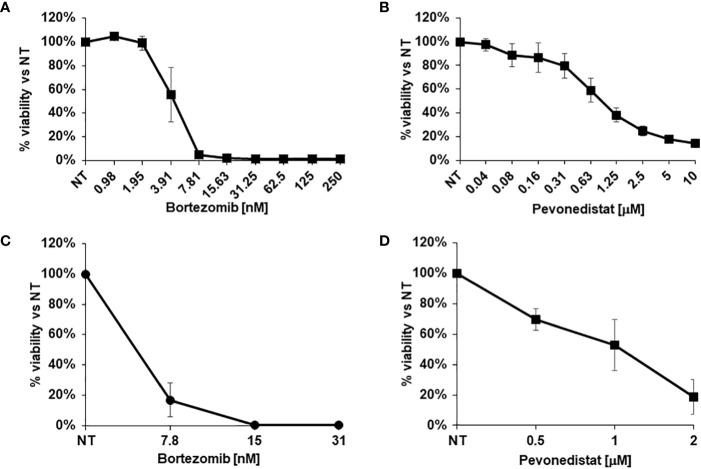
*In vitro* drug response assays. **(A, B)** Viability of 2D cells after *in vitro* treatment with increasing concentrations of Bortezomib **(A)** or Pevonedistat **(B)** for 72h. *C*, **(D)** Viability of 3D organoids after *in vitro* treatment with increasing concentrations of Bortezomib **(C)** or Pevonedistat **(D)** for 1 week. Cell viability was evaluated using the CellTiter-Glo^®^ assay.

**Figure 4:**
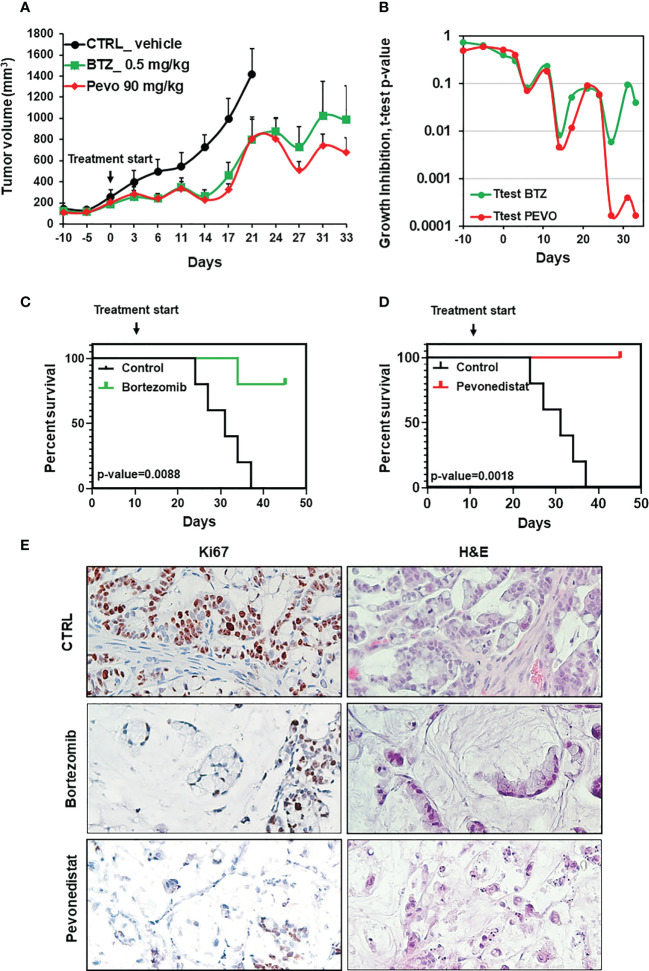
*In vivo* efficacy of pevonedistat and bortezomib. **(A)** *In vivo* growth of three PDX cohorts (n=5), treated for four weeks respectively with vehicle, bortezomib (0.5mg/kg) and pevonedistat (90mg/kg). **(B)** T-test p-values comparing tumor size in the vehicle cohort vs. the bortezomib or pevonedistat cohort, at different treatment times. **(C, D)** Kaplan-Meier survival plots comparing the vehicle cohort vs the bortezomib **(C)** or pevonedistat **(D)** cohorts. P-values are from Longrank test analysis. **(E)** Representative pictures of IHC staining for Ki67 after PDX treatment with vehicle, bortezomib and pevonedistat, at the end of the experiment.

Considering the observed clinical resistance to standard and targeted treatments, and the absence of any therapeutic indication emerging from deep sequencing, the newly derived SR-CRC models were evaluated for sensitivity to the proteasome inhibitor bortezomib and to the NEDD8 inhibitor pevonedistat. Initial assessments on the 2D cell line *in vitro* revealed marked sensitivity to bortezomib, with an IC50 of 4.06 nM (**Figure 3A**) and significant but lower sensitivity to pevonedistat (IC50 = 910 nM, **Figure 3B**). Efficacy of both drugs was confirmed on 3D organoids at one week of treatment, again with higher sensitivity to bortezomib (**Figures 3C, D**). Subsequently, both drugs were tested for efficacy *in vivo*, in the PDX model. Three PDX cohorts were treated for four weeks respectively with vehicle, bortezomib and pevonedistat (see Methods). As showed in **Figure 4A**, both drugs markedly reduced tumor growth compared to the vehicle group, with better efficacy and higher statistical significance for pevonedistat (**Figure 4B**). Kaplan-Meier analysis revealed significantly longer survival of bortezomib and pevonedistat-treated cohorts (log rank *p-value* of 0.009 and 0.002 respectively; **Figures 4C, D**). Intriguingly, while bortezomib seemed more effective *in vitro*, pevonedistat efficacy was slightly more pronounced *in vivo*. Histological analysis of PDX explants at the end of treatment revealed that vehicle-treated tumors not only were larger, but also displayed higher cellularity, while the smaller, bortezomib- and pevonedistat-treated tumors were richer in mucus and necrosis, with lower absolute amounts of Ki67-positive cells (**Figure 4E**). Mucus and necrosis could be at the basis of the oscillations in tumor volume observed in the treated cohorts (**Figure 3A**). Altogether, these results provide preclinical evidence for both drugs as potentially viable therapeutic options for SR-CRC.

## Discussion

Over the last 25 years, the incidence of early onset CRC has been steadily increasing (24). Indeed, substantial increment of CRC incidence in 20-34 year old men and women is estimated to take place by 2030 (25). Early onset CRC is typically diagnosed at an advanced stage and characterized by rapid progression, mucinous or signet ring histology (HGM/SR-CRC) and lower differentiation (26). About 20% of early-onset CRC is hereditary, mostly in the context of the Lynch syndrome, i.e. germline mutations in mismatch repair genes (27). HGM/SR-CRC is associated with higher rate of MSI positivity, a positive predictor of response to checkpoint inhibition-based immunotherapy (28). However, both the mucinous phenotype and Lynch syndrome context are negatively associated with PD-L1 expression by cancer cells in MSI tumors (29). In line with these observations, the patient described here displayed only transient disease stabilization by checkpoint blockade.

Preclinical models like organoids and PDXs are widely recognized as the best possible ways to recapitulate tumor biology and to discover and test new therapeutic strategies (15, 30). This is particularly true when actionable molecular alterations are found, leading to hypothesis-based precision medicine approaches. Unfortunately, the case described here displayed no such alterations, leading to an alternative search for candidate treatments based on previous literature. Accordingly, we tested for sensitivity to proteasome and NEDD8 inhibition in three patient-derived models of increasing complexity: 2D cells, 3D organoids, and *in vivo* PDXs. In this way, drug efficacy could be assessed in multiple experimental conditions, to yield more reliable results. Indeed, the *in vitro* results were highly concordant, with an extremely high efficacy of bortezomib, while the PDX experiments highlighted a therapeutic advantage of pevonedistat. This could be explained by the known limitations of bortezomib efficacy *in vivo* due to high toxicity, poor pharmacokinetics, and low tumor penetration (31, 32). Moreover, the presence of residual mucin is known to form a barrier to drug delivery (33), potentially affecting efficacy of both drugs.

Overall, the preclinical results provided here highlight pevonedistat and bortezomib as potentially effective therapeutic approaches against HGM/SR-CRC. Although limited to a single case, negativity for CDH17 of the tumor and all models further confirmed its potential value as a marker of poor differentiation and pevonedistat sensitivity (18). However, for both drugs, more studies are needed to further improve penetration and response in the context of HGM/SR-CRC. Along this line, a number of studies showed efficacy of combinations of bortezomib or pevonedistat with other drugs. Examples include combination of bortezomib with vorinostat and dexamethasone in relapsed multiple myeloma [NCT01720875 (34)]. Pevonedistat was found to synergize with EGFR pathway inhibition, leading to tumor regression, in CRC xenograft models (35). Bortezomib and pevonedistat could also increase the efficacy of immunotherapy, because both drugs have been shown to induce immunogenic cell death, potentially enhancing antitumor immunity and allowing more durable responses to immunotherapy (36, 37). Additional possibilities for pevonedistat combinations can be derived from its mechanism of action, that ultimately drives stabilization of the replication initiation protein CDT1 at the end of the S-phase. This leads to DNA re-replication, aneuploidy and DNA damage, which in turn results in S and G2/M arrest, causing apoptosis and senescence. For this reason pevonedistat has successfully been tested as a radiosensitizer, in head and neck squamous carcinoma (38), pancreatic and breast cancer (39, 40). Moreover, pevonedistat combination with PARP inhibitors has been described as a possible new strategy for non-small cell lung cancer treatment (41). All the above considerations, together with the better *in vivo* profile, highlight pevonedistat as the preferred candidate for further explorations.

A limitation of this study is that it includes a single case: validation of treatment efficacy and prediction in an adequate cohort of preclinical models is required to move these therapeutic strategies towards clinical assessment in patients. However, this case proves the feasibility and informativeness of the preclinical research strategy in the context of HGM/SR-CRC in early onset patients, where standard therapy frequently fails.

## Data availability statement

The original contributions presented in the study are included in the article and **Supplementary Material**. The raw data supporting the conclusions of this article will be made available by the authors, without undue reservation. Further inquiries can be directed to the corresponding authors.

## Ethics statement

The studies involving human participants were reviewed and approved by Ethics Committee of the Candiolo Cancer Institute. The patients/participants provided their written informed consent to participate in this study. The animal study was reviewed and approved by Istituto Superiore di Sanità. Written informed consent was obtained from the individual(s) for the publication of any potentially identifiable images or data included in this article.

## Author contributions

Conceptualization: EM and ET; experimental design: EM and ET; experimental procedures: ET, CP, FS, RP, MB, MM, EB, and LG; bioinformatic analyses: GCo, GCr, and EB; procurement of models and patient material: EM, SA, and AM; data curation: ET, CP, and EM; writing-original draft preparation: ET and EM; writing-review and editing: EM, ET, CP, CM, SA, and AM; overall supervision and funding acquisition: EM. All authors contributed to the article and approved the submitted version.
