# Supplementary material for: Case report: Preclinical efficacy of NEDD8 and proteasome inhibitors in patient-derived models of signet ring high-grade mucinous colorectal cancer from a Lynch syndrome patient
Source: Front Oncol. 2023 Feb 2;13:1130852. doi: 10.3389/fonc.2023.1130852 (PMC9932521; doi:10.3389/fonc.2023.1130852)
Supplement: Supplementary file 1 [file DataSheet_1.pdf]

Preclinical efficacy of NEDD8 and proteasome inhibitors in patient-derived models of signet ring high-grade mucinous colorectal cancer from a Lynch syndrome patient.

Erica Torchiaro[1], Consalvo Petti[1], Sabrina Arena[1,2], Francesco Sassi[1], Giorgia Migliardi[1], Alfredo Mellano[1], Roberta Porporato[1], Marco Basiricò[1], Loretta Gammaitoni[1], Enrico Berrino[1,3], Monica Montone[1], Giorgio Corti[2], Giovanni Crisafulli [4], Caterina Marchiò[1,3], Alberto Bardelli[1,2,5], and Enzo Medico[1,2]

[1] Candiolo Cancer Institute, Fondazione del Piemonte per l'Oncologia, -IRCCS, Candiolo, Italy.

[2] Department of Oncology, University of Torino, Candiolo, Italy

[3] Department of Medical Sciences, University of Turin, Torino, TO, Italy

[4] IFOM ETS, The AIRC Institute of Molecular Oncology, Milano, Italy

[5] Present address: Department of Oncology, University of Torino, Italy and IFOM ETS – The AIRC Institute of Molecular Oncology, Milan, Italy

Supplementary Methods

Generation and propagation of 2D cell lines

To generate 2D cell lines from tumor tissue and from PDX, the tissue was chop using sterile razor in 10 cm Petri dishes, without media. After that, all material present in the dishes was lysed at 37°C for half one hour using Collagenase 1A 1mg/ml (Sigma- Aldrich SCR103), inverting tubes each 10 minutes. Then the tubed were spin down at 1000rpm for 4 minutes and supernatant discarded. The pellet was suspended in 5 ml of growth media and after 1 minute 4 ml transferred in 10 cm petri dish. This step was repeated for two times and plates incubated over-night a 37°C in 5% CO2. The next day dishes was gently washed and medium changed (DMEM/F12 medium supplemented with 10% fetal bovine serum, 1mM L-glutamine, 100U/ml penicillin and 100ml/ml streptomycin).

Generation and propagation of 3D organoids

Organoids were generated from PDXs generated from colon carcinosis and peritoneal carcinosis. Briefly, specimens were initially smashed in fragments of 2–4 mm in size and then chopped with scalpel for further dissociation in small pieces. After centrifugation, the pellet was washed and centrifuged three times with PBS. At the end of the washing phase, the pellet was resuspended in Basement Membrane Extract (BME; Cultrex BME RGF type 2, Trevigen, Gaithersburg, MD, USA) and 60 µL of organoids-BME suspension was dispensed into the centre of each well of a 24-well plate. After the BME solidify, the tumor organoid medium was added, and organoids were incubated at 37 ◦C and 5% CO2 air incubator. The composition of Tumor Organoid medium is as follows: DMEM/F12+1×B27 supplement (Invitrogen, Carlsbad, CA, USA), 1.25 mM N-acetyl-cysteine (Sigma Aldrich, St. Louis, MO, USA), 1x N-2 supplement (Invitrogen, Carlsbad, CA, USA) 50 ng/mL human EGF (Sigma Aldrich, St. Louis, MO, USA), , Fresh medium was replaced every 2–3 days. Outgrowing organoids were passaged every 10–15 days after mechanical and enzymatic disruption.

In vitro viability assay

To measure cell viability, primary CRC cells and organoids were seeded at different densities (1-10x10^3 cells/well respectively) in 100µl complete growth medium with 10% FBS in 96-well plastic culture plates at day 0. The following day, serial dilutions of Pevonedistat and Bortezomib or DMSO were added to the cells in serum-free medium and incubated for 1 week. After one week of treatments, viability was assessed by ATP-based assay (Cell TITER-Glo Luminescent Cell Viability Assay, Promega). Luminescence was measured with Perkin Elmer Victor 2 (GMI, Ramsey, MN). Data are expressed as average ± SD of three replicates.

Immunohistochemical staining

Formalin-fixed, paraffin-embedded tissues explanted from liver metastatic samples, cell xenografts and PDX were partially sectioned (10 µm thick) using a microtome. 4-µm paraffin tissue sections were dried in a 37°C oven overnight. Slides were deparaffinized in xylene and rehydrated through graded alcohol to water. Endogenous peroxidase was blocked in

3% hydrogen peroxide for 30 minutes. Microwave antigen retrieval was carried out using a microwave oven (750 W for 10 minutes) in 10 mmol/L citrate buffer, pH 6.0. Slides were incubated with monoclonal mouse anti-human Ki67 (1:100; Dako, Glostrup Denmark) overnight at 4°C inside a moist chamber. After washings in TBS, anti-mouse secondary antibody (Dako Envision+System-horseradish peroxidase–labeled polymer, Dako) was added. Incubations were carried out for 1 hour at room temperature. Immunoreactivities were revealed by incubation in DAB chromogen (DakoCytomation Liquid DAB Substrate Chromogen System, Dako) for 10 minutes. Slides were counterstained in Mayer's hematoxylin, dehydrated in graded alcohol, cleared in xylene, and the coverslip was applied by using DPX. A negative control slide was processed with secondary antibody, omitting primary antibody incubation. For CDH17 IHC evaluation, we employed CDH17 monoclonal antibody (M01), clone 1H3 (Abnova Taipei, Taiwan) following the manufacturer protocol.

Targeted NGS sequencing

The OCAv3 target gene panel was used to assess mutation status of 161 genes in the primary tumor of the patients and in the derived primary cell line, organoid and PDX. A total of 40 ng of DNA was subjected to library preparation using the Ion AmpliSeq Library kit Plus (Thermo Fisher Scientific) following the manufacturer's instructions.

Library were sequenced to the Ion GeneStudio S5 Plus System for the sequencing (Thermo Fisher Scientific) for an expected mean read depth of 500×.

BAM files derived from processed raw data were generated by the Ion Torrent platform-specific pipeline software (Torrent Suite Software 5.12, Thermo Fisher Scientific) and analyzed by the Oncomine OCAv3 w3.0–DNA–Single Sample (version. 5.10) on the Ion Reporter Software (version 5.10.5.0) (Thermo Fisher Scientific). The Ion Reporter workflow was applied to identify SNVs, indels and CNVs with a tumor-only pipeline using the parameters and the Boolean chain reported here PMID: 35260767.

Whole exome sequencing

We used the peripheral blood mononucleated cells and fresh tissues available for CRC-1731 patient. Libraries were prepared using Truseq Rapid Exome kit (Illumina), according to the manufacturer's protocol. We started from 150 ng of gDNA and it was then fragmented using transposons and simultaneously were added adaptor sequences. After this step, purified tagmented DNA was amplified and were added the index adapters. To assess the

size distribution of the DNA fragments, it was used a 2100 Bioanalyzer with High Sensitivity DNA Assay Kit (Agilent Technologies). Subsequently, DNA libraries containing unique indexes were combined into a single pool and targeted regions of the DNA were binded with capture probes. In the last step, the enriched library was amplify and sequenced on the Illumina NextSeq500 sequencer (Illumina) generating 150 bp paired end reads.
All FASTQ files were mapped to the human reference genome (hg38 version) using BWA (1), and PCR duplicates were filtered out by Picard's MarkDuplicates command (Broad Institute. Picard Tools. Available at: https://broadinstitute.github.io/picard/. 2019. Accessed: April 5, 2019). Then we made use of the tools described in Corti et al. 2019 (2) to call somatic and germline single nucleotide variants, copy-number variations and INDELs.
Acquired microsatellite unstable regions was identified as reported in published literature (3, 4). Using the human reference genome, homopolimer and microsatellite genomic regions were identified and reads located in one of the identified regions were extracted for two matched samples (PBMC and Tumor), discarding regions with less than 20 encompassing reads. Read lengths of normal and tumor samples were measured (at least 25,000 regions for each pair were included in the analysis), and the distribution for each sample pair was calculated. Next, a standard $\chi 2$ test was performed for each region and the fractional abundance of unstable regions was defined as the ratio between the number of unstable regions (*p-value* <0.05) and the total number of regions included in the analysis.

Animal studies

To generate PDXs (Patient-Derived Xenografts), tumor carcinosis from colon and peritoneum were subcutaneously implanted in the right flank of 7-week-old NOD-SCID mice (Charles River Laboratory, Wilmington, MA, USA). To test the response to Pevonedistat and Bortezomib the organoids derived from PDX of colon carcinosis were subcutaneously implanted in the right flank of 7-week NOD-SCID mice and when PDX was established the tumor was propagated in a cohort of 24 mice. Established tumors of the most homogeneous in size (average volume 250 mm^3) were randomized in three group: one group of 5 control mice; one group of 5 mice treated with bortezomib at 0.5 mg/kg for three times a week intraperitoneal for 5 weeks; one group of 5 mice treated with pevonedistat (twice per day, on days 1, 4, 8, 11, 15, 18, 22, 25, 29, 32) at 90 mg/kg of drug, administered subcutaneously. *In vivo* bortezomib and pevonedistat treatment protocols were defined based on previous literature (5)(6). Tumor size was evaluated twice per week by caliper measurements and the approximate volume of the mass was calculated using the formula $4/3\pi \cdot (d/2)^2 . D/2$, where d

is the minor tumor axis and *D* is the major tumor axis. The protocol was reviewed by the local Animal Welfare Body (OPBA) of the Candiolo Cancer Institute IRCCS (Candiolo, Italy) and authorized by Italian Ministry of Health (Licence Number No. 195/2015-PR), according to the Italian Law on the protection of animals used for scientific purposes (DLgs 26/2014) which enforces the EU Directive 63/2010.

Statistical Analysis

Data are represented as mean ± standard deviation (SD) or standard error (SEM) of biological triplicates. Comparisons of drug efficacy among groups were performed using one-way ANOVA followed by Student's t-test for unpaired samples. $p \leq 0.05$ was considered significant.

References

1. Li H. Aligning sequence reads, clone sequences and assembly contigs with BWA-MEM. In: Li H, editor.: arXiv:1303.3997v2 [q-bio.GN]; 2013.
2. Corti G, Bartolini A, Crisafulli G, Novara L, Rospo G, Montone M, et al. A Genomic Analysis Workflow for Colorectal Cancer Precision Oncology. Clin Colorectal Cancer. 2019;18(2):91-101.e3.
3. Niu B, Ye K, Zhang Q, Lu C, Xie M, McLellan MD, et al. MSIsensor: microsatellite instability detection using paired tumor-normal sequence data. Bioinformatics. 2014;30(7):1015-6.
4. Russo M, Crisafulli G, Sogari A, Reilly NM, Arena S, Lamba S, et al. Adaptive mutability of colorectal cancers in response to targeted therapies. Science. 2019;366(6472):1473-80.
5. Tsumagari K, Abd Elmageed ZY, Sholl AB, Green EA, Sobti S, Khan AR, et al. Bortezomib sensitizes thyroid cancer to BRAF inhibitor. Endocr Relat Cancer. 2018;25(1):99-109.
6. Picco G, Petti C, Sassi F, Grillone K, Migliardi G, Rossi T, et al. Efficacy of NEDD8 Pathway Inhibition in Preclinical Models of Poorly Differentiated, Clinically Aggressive Colorectal Cancer. J Natl Cancer Inst. 2017;109(2).
