# Supplementary material for: Case report: Preclinical efficacy of NEDD8 and proteasome inhibitors in patient-derived models of signet ring high-grade mucinous colorectal cancer from a Lynch syndrome patient
Source: Front Oncol. 2023 Feb 2;13:1130852. doi: 10.3389/fonc.2023.1130852 (PMC9932521; doi:10.3389/fonc.2023.1130852)
Supplement: Supplementary file 2 [file Table_1.pdf]

ID	gene	# locus hg38	type	length	genotype	depth	VAF	function	codon	exon	protein	coding	sift	polyphen	grantham
Tumor Tissue	**CTNNB1**	chr3:41224642-41224643	SNV	1	C/T	1216	**0.07**	missense	TTT	3	p.Ser45Phe	c.134C>T	0.0	0.996	155.0
	**KRAS**	chr12:25245345-25245346	SNV	1	C/T	889	**0.05**	missense	GAC	2	p.Gly13Asp	c.38G>A	0.0	0.803	94.0
	**GNAS**	chr20:58909366-58909367	SNV	1	G/A	836	**0.04**	missense	CAT	8	p.Arg201His	c.602G>A	0.0	1.0	29.0
PDX	**CTNNB1**	chr3:41224642-41224643	SNV	1	C/T	1450	**1**	missense	TTT	3	p.Ser45Phe	c.134C>T	0.0	0.996	155.0
	**KRAS**	chr12:25245345-25245346	SNV	1	C/T	1993	**0.49**	missense	GAC	2	p.Gly13Asp	c.38G>A	0.0	0.803	94.0
	**GNAS**	chr20:58909366-58909367	SNV	1	G/A	350	**0.48**	missense	CAT	8	p.Arg201His	c.602G>A	0.0	1.0	29.0
	**ARID1A**	chr1:26779342-26779343	INDEL	1	AG/A	1125	**0.18**	frameshiftDeletion	TCG	20	p.Ile1816fs	c.5445delG			
	**ERBB2**	chr17:39725364-39725365	SNV	1	G/A	441	**0.1**	missense	CAC	22	p.Arg896His	c.2687G>A	1.0	0.0	29.0
3D Organoids	**CTNNB1**	chr3:41224642-41224643	SNV	1	C/T	257	**1**	missense	TTT	3	p.Ser45Phe	c.134C>T	0.0	0.996	155.0
	**KRAS**	chr12:25245345-25245346	SNV	1	C/T	1222	**0.66**	missense	GAC	2	p.Gly13Asp	c.38G>A	0.0	0.803	94.0
	**GNAS**	chr20:58909366-58909367	SNV	1	G/A	336	**0.52**	missense	CAT	8	p.Arg201His	c.602G>A	0.0	1.0	29.0
2D Cells	**CTNNB1**	chr3:41224642-41224643	SNV	1	C/T	868	**1**	missense	TTT	3	p.Ser45Phe	c.134C>T	0.0	0.996	155.0
	**KRAS**	chr12:25245345-25245346	SNV	1	C/T	1991	**0.66**	missense	GAC	2	p.Gly13Asp	c.38G>A	0.0	0.803	94.0
	**GNAS**	chr20:58909366-58909367	SNV	1	G/A	1269	**0.49**	missense	CAT	8	p.Arg201His	c.602G>A	0.0	1.0	29.0

Supplementary Table 1. Deep sequencing of a 161-gene panel (ThermoFisher Oncomine Comprehensive Assay v.3) comparing germline DNA against tumor (from third surgery) or its derivatives, with variant allele frequency (VAF) for somatic mutation detection threshold at 0.02.
